# Calendar Aging Triggers Critical Performance Degradation of Lithium Primary Batteries

**DOI:** 10.1007/s40820-026-02354-x

**Published:** 2026-11-05

**Authors:** Mengjie Li, Shuzhi Zhao, Fei Wei, Wen Jia, Zi-Feng Ma

**Affiliations:** 1 Shanghai Electrochemical Energy Devices Research Center, School of Chemistry and Chemical Engineering, Shanghai Jiao Tong University, Shanghai, 200240 People’s Republic of China; 2 Shaanxi Applied Physics Chemistry Research Institute, Xi’an, 710061 People’s Republic of China; 3 College of Smart Energy, Shanghai Jiao Tong University, Shanghai, 200240 People’s Republic of China

**Keywords:** Lithium primary cells, Calendar aging, Performance degradation, Interfaces evolution

## Abstract

Lithium primary cells have garnered significant attention owing to long storage life and high safety. However, the capacity degradation law derived from accelerated aging methods is not universally applicable to all operating conditions; thus, lifetime evaluation under calendar aging poses substantial challenges while holding remarkable significance. Herein, comprehensive characterizations were conducted on lithium primary cells subjected to 10-year calendar aging, which aimed to gain a holistic understanding of the degradation mechanism. The results reveal that the surface of the postmortem electrode is covered by complex interfacial species, which exhibit a dual role: They contribute to the low self-discharge rate of cells, and paradoxically, the further deposition of these species at the interfaces between active materials and conductive agents significantly increases the interfacial impedance of electrode, thereby deteriorating the C-rate performance of the cells. This work provides novel insights for the design and development of lithium primary cells.

**Supplementary Information:**

The online version contains supplementary material available at https://doi.org/10.1007/s40820-026-02354-x.

## Highlights

- Cell data from a decade of calendar aging were investigated.
- The high C-rate performance of the cell is severely attenuated after calendar aging.
- The interface between electrode and electrolyte was analyzed.
- The type of ion fragment points to the serious degradation of the electrolyte system.

**Supplementary Information:**

The online version contains supplementary material available at https://doi.org/10.1007/s40820-026-02354-x.

## Graphical abstract

**Figure:**
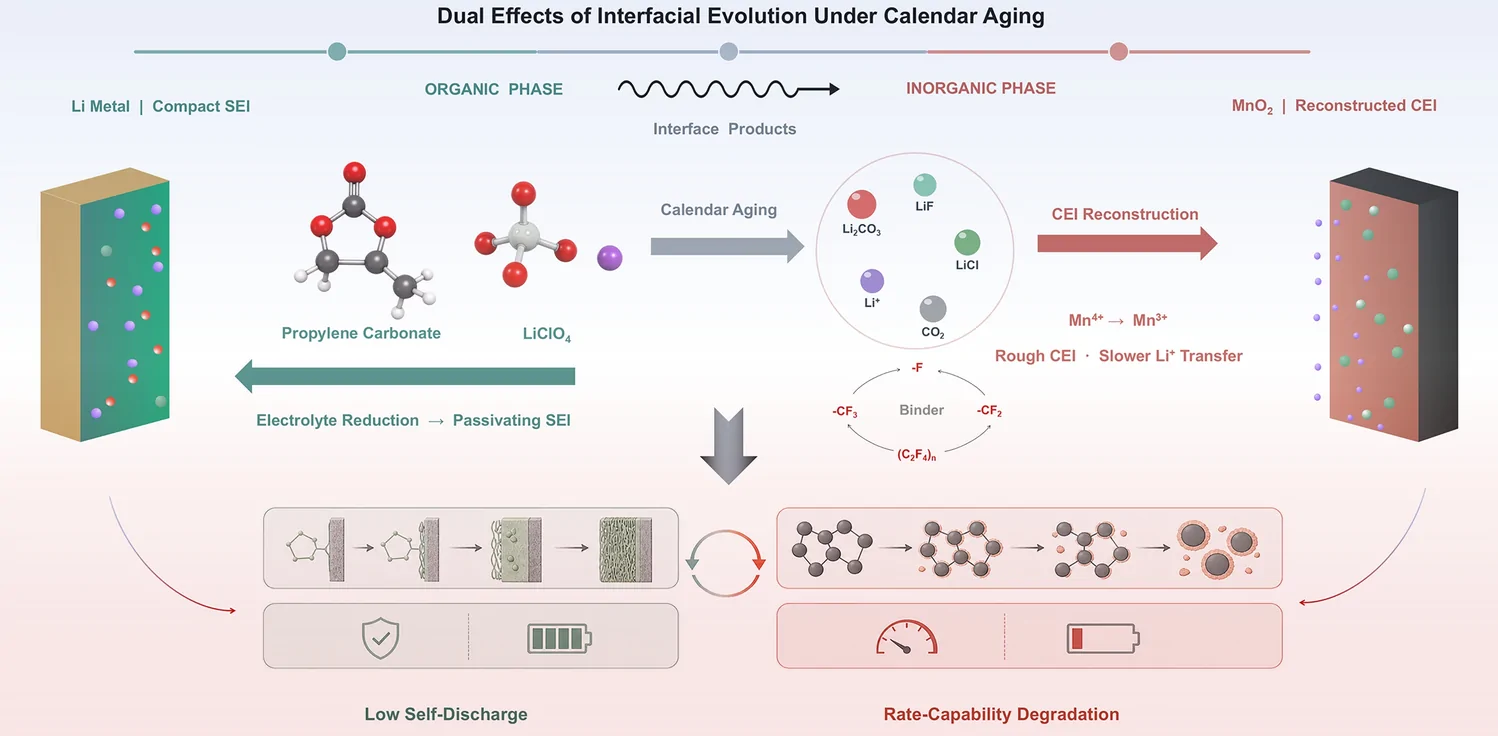

## Introduction

Lithium manganese dioxide (Li/MnO_2_) cells, as one of the most widely utilized lithium primary cell systems, are appreciated for the high specific energy and low self-discharge rates, and are employed in a variety of applications including medical devices, portable equipment, underwater identification, and communication reconnaissance for both civilian and military purposes [1–6]. Since the commercialization by Sanyo Corporation, the electrochemical behavior of Li/MnO_2_ cells has been extensively investigated, encompassing performance enhancement and analysis of discharge mechanisms [7–10]. Storage life is a critical attribute of interest to users of Li/MnO_2_ cells, with a theoretical annual self-discharge rate of less than 1% and a potential storage duration of up to 20 years. Regrettably, in practical use, cells exhibit varying levels of capacity degradation due to differences in materials, design methodologies, and manufacturing processes. To rapidly acquire datasets for different storage durations, researchers often subject cells to high temperatures and employ the Arrhenius model to equivalently simulate calendar storage. However, the complex electrochemical reaction processes are not solely dependent on temperature [11–14]. The Arrhenius model, which is widely used for accelerated aging prediction, assumes a single dominant degradation mechanism with a constant activation energy across all temperatures and time scales [15, 16]. This fundamental assumption leads to significant discrepancies when extrapolating short-term high-temperature data to long-term room-temperature conditions. Reference based on several years calendar aging data has demonstrated that battery degradation exhibits distinct nonlinear characteristics, including threshold effects and mechanism transitions, which cannot be captured by the traditional Arrhenius model [11]. Specifically, elevated temperatures may activate additional degradation pathways that do not occur under natural storage conditions, while the coupling effects between multiple degradation factors become increasingly pronounced over extended time periods [17, 18].

Calendar aging refers to the degradation of performance due to internal chemical reactions occurring in the absence of externally applied current, and the associated data are challenging to collect due to factors such as time and cost [19–23]. The US Advanced Battery Consortium (USABC) emphasizes that calendar aging should be given equal consideration as cycle life [24]. References have increasingly focused on the self-discharge behavior of lithium-ion cells and have attributed the observed results primarily to the dissolution of transition metals, interface instability, and chemically induced mechanical effects [25–36]. As a reference, electrode structure engineering strategies such as pressurized organic electrode design have been shown to effectively improve interface stability and actual performance of batteries under extreme service conditions [37]. However, unlike lithium-ion cells, lithium primary cells, including Li/MnO_2_ cells, do not require consideration of the cycle life under calendar aging conditions. The capacity, C-rate capability, and internal resistance of cells after prolonged storage are key performance indicators that are closely monitored, as the metrics are strongly correlated with changes at the interfaces of the anode, cathode, and electrolyte. On the anode side, Li_2_CO_3_ (derived from solvent reaction) and Mn element (derived from cathode elution) on the surface of lithium metal will increase the internal resistance under high temperature accelerated simulated aging. An effective measure to alleviate this phenomenon is to form a solid interface film on the electrode surface through pre-discharge. On the electrolyte side, the commercial solvent propylene carbonate (PC) contained in the electrolyte decomposes under acidic or alkaline conditions, producing gases that can lead to the failure of lithium manganese pouch cells over long-term storage. On the cathode side, the *γ*/*β* mixed-phase MnO_2_ is commonly used in lithium primary cells due to its superior electrochemical properties. However, it is inevitable that the structure of the transition metal oxide will be damaged after long-term storage due to the Jahn–Teller effect or the influence of lithium-ion insertion [38, 39].

Upon recognizing the significance of understanding the degradation mechanisms associated with calendar aging in cells, we have maintained records of previously manufactured Li/MnO_2_ pouch cells and utilized increasingly advanced characterization techniques to study the calendar aging. A systematic examination was conducted on cells that had been stored for fresh, 3, 6, 8, and 10 years, assessing the time-dependent changes in internal resistance, C-rate capability, and specific capacity. The electrodes from cells subjected to calendar aging were dissected and deeply analyzed to elucidate the degradation mechanisms across different time scales. These valuable data lay the theoretical foundation for the development and enhancement of storage technologies for lithium primary cells.

## Experimental Section

### Fabrication of Li-MnO_2_ Primary Pouch Cells

Cathode electrodes were fabricated by preparing a homogeneous slurry containing manganese dioxide (MnO_2_, active material), Super P (conductive carbon black), and polytetrafluoroethylene (PTFE, binder) at a mass ratio of 90%:5%:5%. The slurry was uniformly cast onto aluminum mesh current collectors, followed by drying at 200 °C for 4 h under vacuum to ensure complete removal of excess free water. Lithium metal foil served as the anode electrode. Cells were assembled via a typical winding process and then encapsulated in aluminum plastic film. The electrolyte (1.0 M LiClO_4_) dissolved in a mixed solvent of propylene carbonate (PC) and dimethoxyethane (DME) at a volume ratio of 7:3, which was injected into the sealed pouches. After vacuum evacuation to eliminate residual air, the excess packaging material was trimmed. A pre-discharge step was subsequently conducted at a constant current density of 0.1 C to stabilize the electrode–electrolyte interface and inhibit the initial Mn elution. The pre-discharge ratio is 3% of the nominal capacity. All cells designated for calendar aging were fabricated following the exact same procedure outlined above to eliminate batch-to-batch variations, ensuring the reliability of subsequent storage performance comparisons. Detail battery information, storage instructions, and characterization methods are shown in supplementary materials.

## Results and Discussion

### Electrochemical Performance Evaluation

**Fig. 1:**
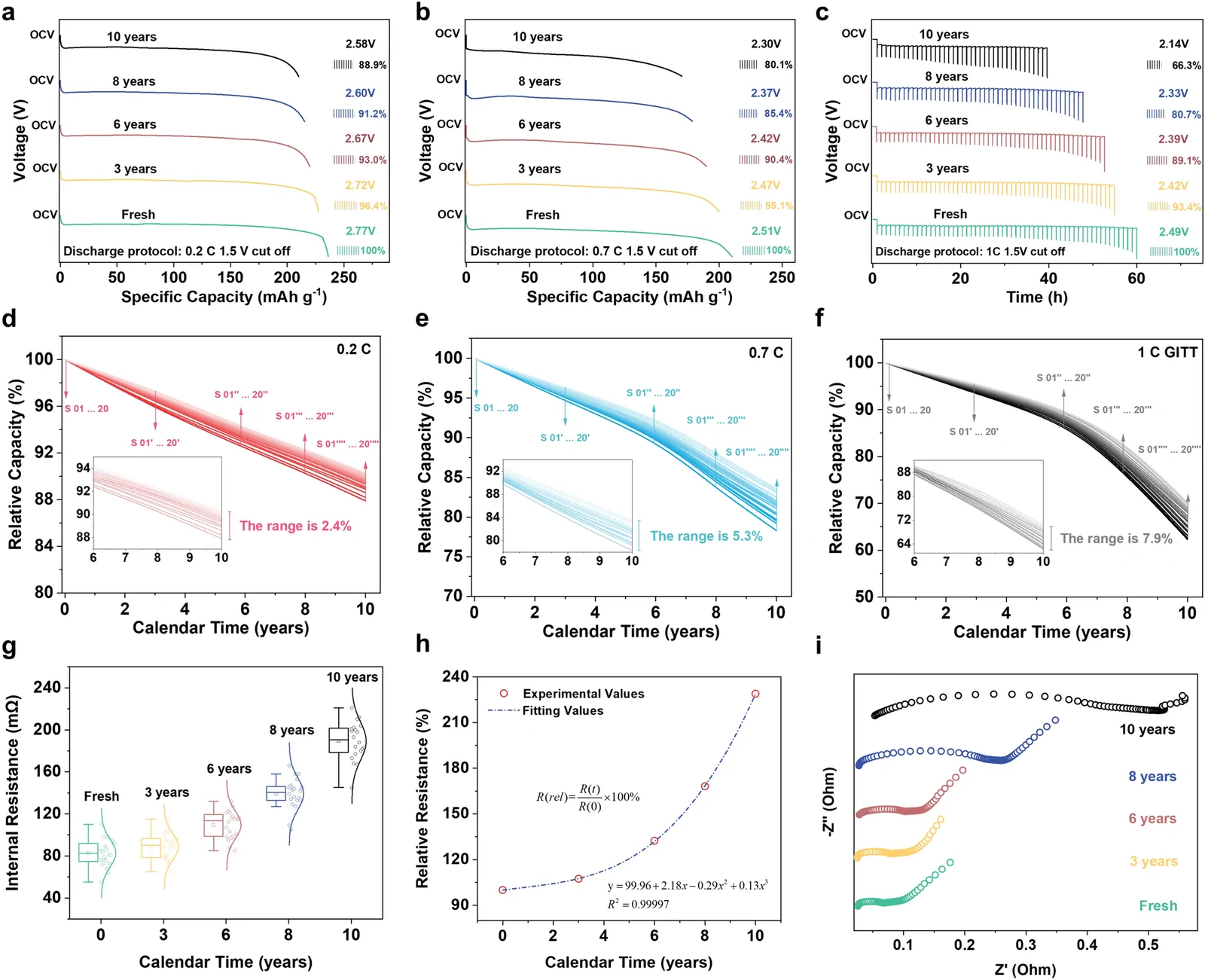
Electrochemical performance of cells with different calendar time. The discharge curves of cells with different calendar time at **a** 0.2 C, **b** 0.7 C, **c** 1 C GITT. Relative capacity versus calendar time at **d** 0.2 C, **e** 0.7 C, **f** 1 C GITT. **g** Internal resistance versus calendar time curves. **h** Fitting curve of relative resistance versus calendar time. **i** EIS Nyquist plots of cells with different calendar time

Twenty cells were randomly selected for each calendar aging interval to investigate the capacity variation of cells under different storage durations. The discharge capacities of fresh cells and those aged for different years gradually decreased at a low current density of 0.2 C, with a simultaneous reduction in the corresponding voltage plateau (Fig. 1a–c). These degradation phenomena conform to the typical Arrhenius relationship, and the results show that the average annual self-discharge rate is approximately 1.2%. In contrast, when the cells were discharged at 0.7 C, the decline in capacity retention rate and voltage plateau exhibited a more pronounced trend. The capacity retention rate of the cells dropped to 80.1%, and the corresponding voltage plateau decreased to 2.30 V after 10 years of storage. Under the GITT discharge protocol at 1 C, the degradation phenomenon was completely unexpected: The capacity retention rate severely attenuated to 66.3%, with the corresponding plateau voltage only reaching 2.14 V. This severe electrochemical degradation indicates that the cells have basically lost the inherent output performance. Furthermore, the phenomenon of deviating from the Arrhenius temperature dependence may lead to incorrect estimation of the cell degradation state. To clearly evaluate the results of these electrochemical performance changes, statistical comparisons were conducted on the twenty samples under each discharge protocol (Fig. 1d–f). The performance degradation of the cells under low C-rate showed an almost linear relationship, while the performance declined sharply with the increase in applied load, especially when the storage duration exceeded 6 years. It is worth noting that the consistency of the capacity retention rate of initial samples usually depends on the manufacturer’s production level; however, the consistency becomes abnormally variable after storage. The range of capacity retention rates among the 10-year stored samples reached 7.9% at high C-rate discharge.

Figure 1g shows the variation trend of internal resistance with different calendar aging durations. The average internal resistance values of fresh cells, and those stored for 3, 6, 8, and 10 years are 82.7, 88.8, 109.5, 139.0, and 189.3 mΩ, respectively, with a relative change rate of internal resistance as high as 228.8%. Electrochemical impedance spectroscopy (EIS) was used to evaluate the effect of storage time on bulk resistance and interfacial impedance. In the Nyquist plot of Fig. 1i, the semicircle and the inclined line observed in the high-frequency and low-frequency regions of the EIS are assigned to the charge-transfer resistance (R_ct_) and Warburg impedance (Z_w_), respectively. The ohmic resistance (R_s_), represented by the high-frequency intercept on the real impedance axis, increases monotonically with extended storage time. During early storage, R_s_ increases modestly as a result of gradual electrolyte decomposition and mild passivation film thickening. With prolonged aging, substantial buildup of insulating by-products lowers electrolyte ionic conductivity and raises interfacial contact resistance, causing a pronounced acceleration in R_s_ growth. Fresh cells exhibit the smallest charge-transfer resistance compared with those after calendar aging. The variation of the impedance data in the figure implies that in the early stage of storage, the interfacial reaction forms impedance at a slow rate, which slightly hinders electron transfer and results in a small increase in R_ct_. After 6 years of storage, the accumulation of high-impedance by-products intensifies, significantly hindering electron transfer across the interface and further leading to a lack of reaction sites, which causes a sharp increase in R_ct_. The EIS results confirm the capacity degradation regulation in terms of the change in interfacial impedance.

### Postmortem Cathode Electrode Interface Morphology and Chemical State Evolution

**Fig. 2:**
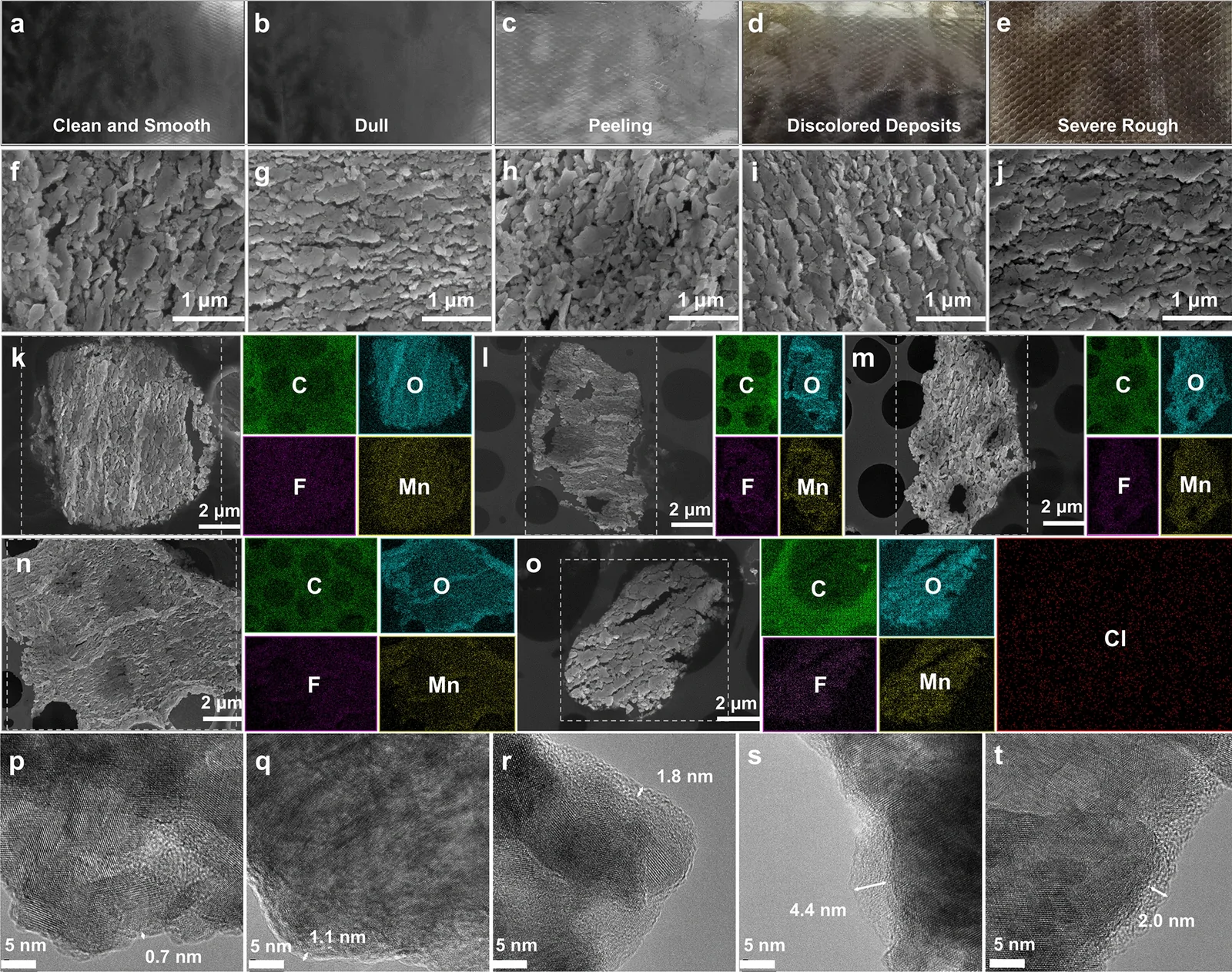
Structure characterization of postmortem cathode electrode with different calendar time. The photographs of postmortem cathode electrode at stage of **a** fresh, **b** 3 years, **c** 6 years, **d** 8 years, **e** 10 years. SEM and EDS images of **f, k** fresh, **g, l** 3-year, **h, m** 6-year, **i, n** 8-year, **j, o** 10-year postmortem cathode electrode. TEM images of **p** fresh, **q** 3-year,** r** 6-year, **s** 8-year, **t** 10-year postmortem cathode electrode

Figure 2a–e presents the photographs of postmortem cathode electrode at different storage stages. Postmortem cathode of fresh cell maintains the flatness and smoothness after rolling process. After 6 years of storage, slight surface peeling occurs, subsequently, the surface discolors, the amounts of deposits increase, and surface roughness is aggravated. Scanning electron microscopy (SEM) was employed to investigate the microstructural integrity and elemental distribution of the postmortem cathode sheets. During the first 6 years of calendar aging, the cathode microstructure remains well-preserved, with the conductive agent Super P dispersed at the interfaces between MnO_2_ particles. No particle agglomeration or cracking is observed, indicating that the cathode framework exhibits stable mechanical properties supported by the binder (Fig. 2f–j). Notably, as the storage duration prolonged, the surface smoothness of the cathode slightly decreases, and the edges of MnO_2_/Super P aggregates become blurred.

Energy-dispersive spectrometer (EDS) was used to track the elemental distribution of the cathode across random cross sections (Fig. 2k–o). Throughout 10-year storage period, the cathode surface is dominated by O, F, and Mn elements, with no significant changes in the relative contents. The stable distribution of these major elements indicates that the storage-induced reaction is strictly limited to the nanoscale cathode electrolyte interface and does not alter the bulk phase composition of the cathode. A crucial result from EDS analysis is that the signal of Cl is only detected at the cathode stored for 10 years, which is attributed to the fact that Cl initially exists as ClO_4_^−^ dissolved in the liquid electrolyte, with minimal adsorption on the cathode surface. Subsequently, the intensification of electrolyte decomposition leads to the accumulation of Cl-containing species on the cathode surface. High-resolution transmission electron microscopy (HR-TEM) was utilized to resolve the interfacial structure of the postmortem electrode. For fresh and 3-year postmortem electrode, HR-TEM images (Fig. 2p–t) show a clear and sharp interface between *γ*/*β*-MnO_2_ and the surrounding region, with no discrete interfacial layer observed, and the lattice fringes of the active material are distinguishable. As the storage duration extends, the organic layer on the surface of MnO_2_ increases first and then decreases, suggesting that there may be a secondary transformation of the surface species, which is related to the irreversible degradation of the interface.

**Fig. 3:**
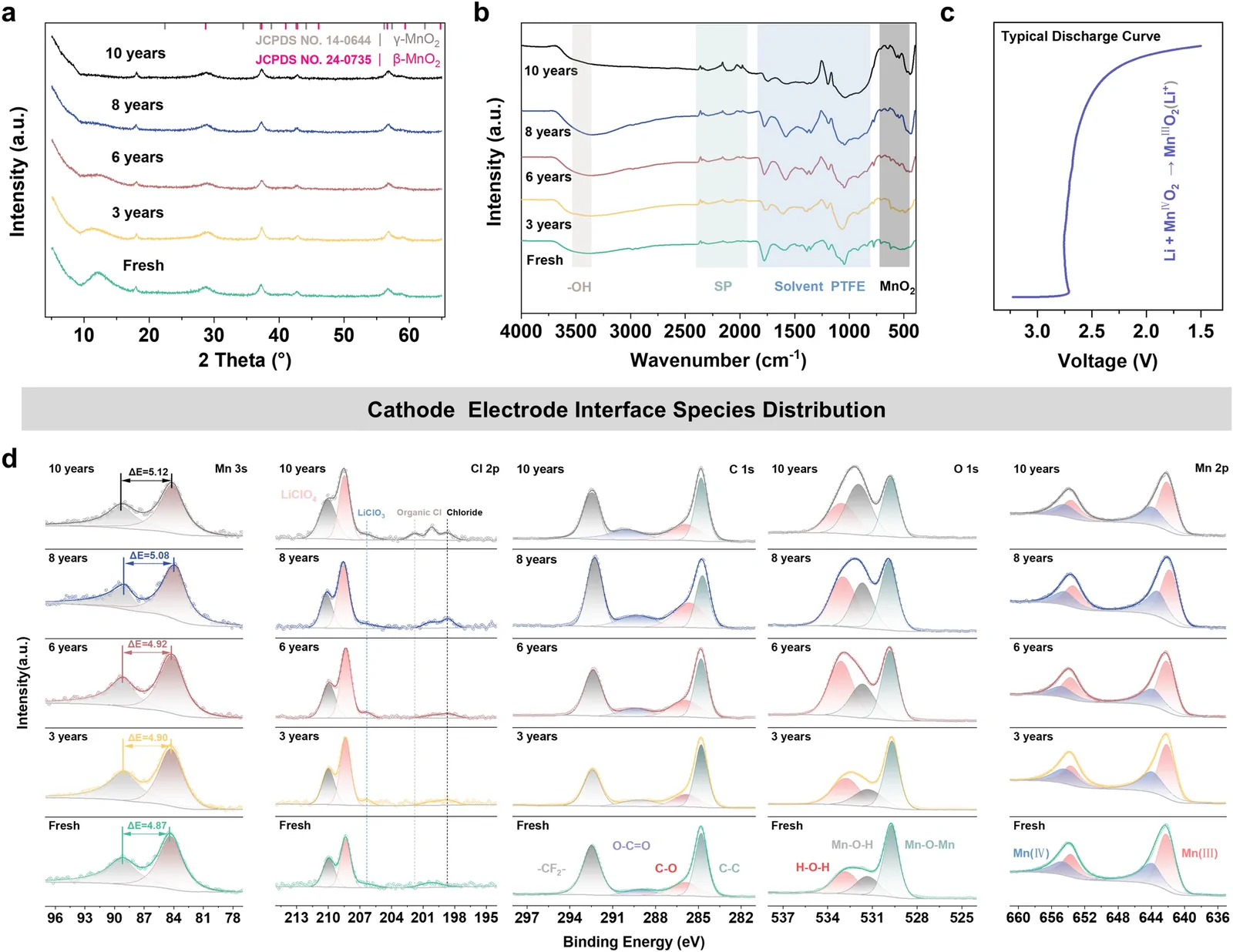
Characterization of physical and chemical properties to identify structural differences under different calendar time. **a** XRD pattern of postmortem cathode electrode of different calendar time. **b** FTIR spectra of postmortem cathode electrode of different calendar time. **c** Typical discharge curve of Li-MnO_2_ battery. **d** Mn 3*s*, Cl 2*p*, C 1*s*, O 1*s*, and Mn 2*p* XPS spectra of postmortem cathode electrode of different calendar time

X-ray diffraction (XRD) patterns of postmortem cathode with different storage durations are shown in Fig. 3a, where characteristic peaks were observed at 2*θ *= 11.9°, 18.0°, 28.7°, 37.1°, 42.7°, and 56.7°. The characteristic peaks at 28.7°, 37.1°, 42.6°, and 56.7° are assigned to *γ*/*β* mixed-crystal MnO_2_ after subtracting the individual XRD backgrounds of each component [40, 41]. During the storage process, the peak at 11.9° gradually disappears, which is attributed to the coverage of conductive carbon black by insulating by-products generated from electrolyte decomposition and the subsequent disruption of the electron conduction network. The relative intensities of the characteristic peaks at 28.7°/37.1° first decrease and then increase, which may be associated with the lattice distortion of MnO_2_ induced by Li^+^ intercalation and the subsequent bulk reconstruction of MnO_2_. In addition, there is no significant change in the number or relative intensity of the MnO_2_ diffraction peaks, indicating that the active material retains its bulk framework without dissipation. This observation is consistent with the stable self-discharge behavior of the cells at low current density. Fourier transform infrared (FTIR) spectra of postmortem cathode with different storage durations were used to characterize the structure and composition of substances generated during the storage process (Fig. 3b). The –OH peak corresponding to 3438 cm^−1^ first increases in intensity and then decreases, which is attributed to unavoidable moisture introduced during electrode manufacturing in the cathode [42]. The peak weakens as the moisture is consumed by reactions occurring on both the electrolyte and cathode sides in the later storage stage. The peaks at 1767, 1588, and 1379 cm^−1^ exhibit fluctuations in the middle storage stage, and the 1767 cm^−1^ peak corresponds to the C=O stretching vibration of the electrolyte solvent, while the 1588 and 1379 cm^−1^ peaks are assigned to the asymmetric and symmetric COO^−^ stretching vibrations of electrolyte decomposition products, respectively. For the peak at 1053 cm^−1^ (corresponding to C–O–C stretching vibration), its shape gradually broadens, which is attributed to the accumulation of diverse C–O-containing organic by-products. The infrared absorption at 582 cm^−1^ is assigned to the Mn-O stretching vibration of MnO_6_ octahedral units, corresponding to MnO_2_ with bound water, and this absorption gradually diminishes with prolonged storage. The characteristic peak at 442 cm^−1^ gradually becomes sharper and shows good consistency with the characteristic band of typical *β*-MnO_2_, indicating that the active material is mainly transformed into this crystal form. The FTIR results preliminarily indicate that the primary cause for the sharp decline of capacity at high current density during the storage process is the formation of an insulating by-product layer.

X-ray photoelectron spectroscopy (XPS) was employed to investigate the composition and chemical state of postmortem cathode (Fig. 3d). The splitting of the Mn 3*s* peak originates from the parallel spin coupling between electrons in the 3*s* and 3*d* orbitals [43]. The splitting energy of the corresponding orbital gradually increases from 4.87 to 5.12 eV during storage, with the average oxidation state (AOS) of Mn decreasing from 3.47 to 3.19. This reduction of Mn^4+^ to Mn^3+^ is strictly confined to the cathode surface, as XPS has an effective sampling depth of ~ 5.4 nm for Mn 2*p* photoelectrons (3 times the inelastic mean free path of ~ 1.8 nm for MnO_2_ under Al Kα irradiation) [44]. The Mn 2*p* orbital was deconvoluted into peaks corresponding to Mn^3+^ and Mn^4+^, and the ratio of peak areas fluctuates slightly, indicating that the bulk structure of MnO_2_ is maintained. The initial presence of Mn^3+^ is caused by pre-discharge treatment during electrode manufacturing, which is implemented to prevent Mn elution. The O 1*s* spectrum can be deconvoluted into three components: The low binding energy (BE) peak at 529.7 eV corresponds to Mn-O-Mn (lattice oxygen), and the slight intensity decrease is associated with minor lattice reconstruction [45]. The medium BE peak at 531.3 eV is assigned to Mn-O-H, the intensity increases continuously, and the BE gradually shifts to 531.9 eV (corresponding to Mn-O-Li), which is due to the reduced electron cloud density of O atoms caused by Li^+^ substituting for H^+^. The high BE peak at 532.8 eV belongs to H–O–H (adsorbed water), and its intensity first increases then decreases, which implies the gradual disappearance of the moisture-rich environment in the cathode. A typical change in the deconvoluted C 1*s* spectrum is that the intensity of the peak at 289.2 eV (corresponding to O–C=O) increases continuously, with its BE shifting to 290.0 eV, indicating a change in the chemical environment of the degradation products. In the Cl 2*p* spectrum, the intensities of the peaks at 210.0 eV (ClO_4_^−^) and 208.3 eV (ClO_3_^−^) first increase then decrease, which is related to the initial enrichment of LiClO_4_ at the cathode followed by its gradual decomposition. Additionally, an increasing number of new peaks emerge in the range of 198.8–201.0 eV, corresponding to organic chlorides and metal chlorides, which will inevitably damage the intrinsic electrical conductivity of the cathode [46, 47].

### Postmortem Anode Electrode Interface Morphology and Chemical State Evolution

**Fig. 4:**
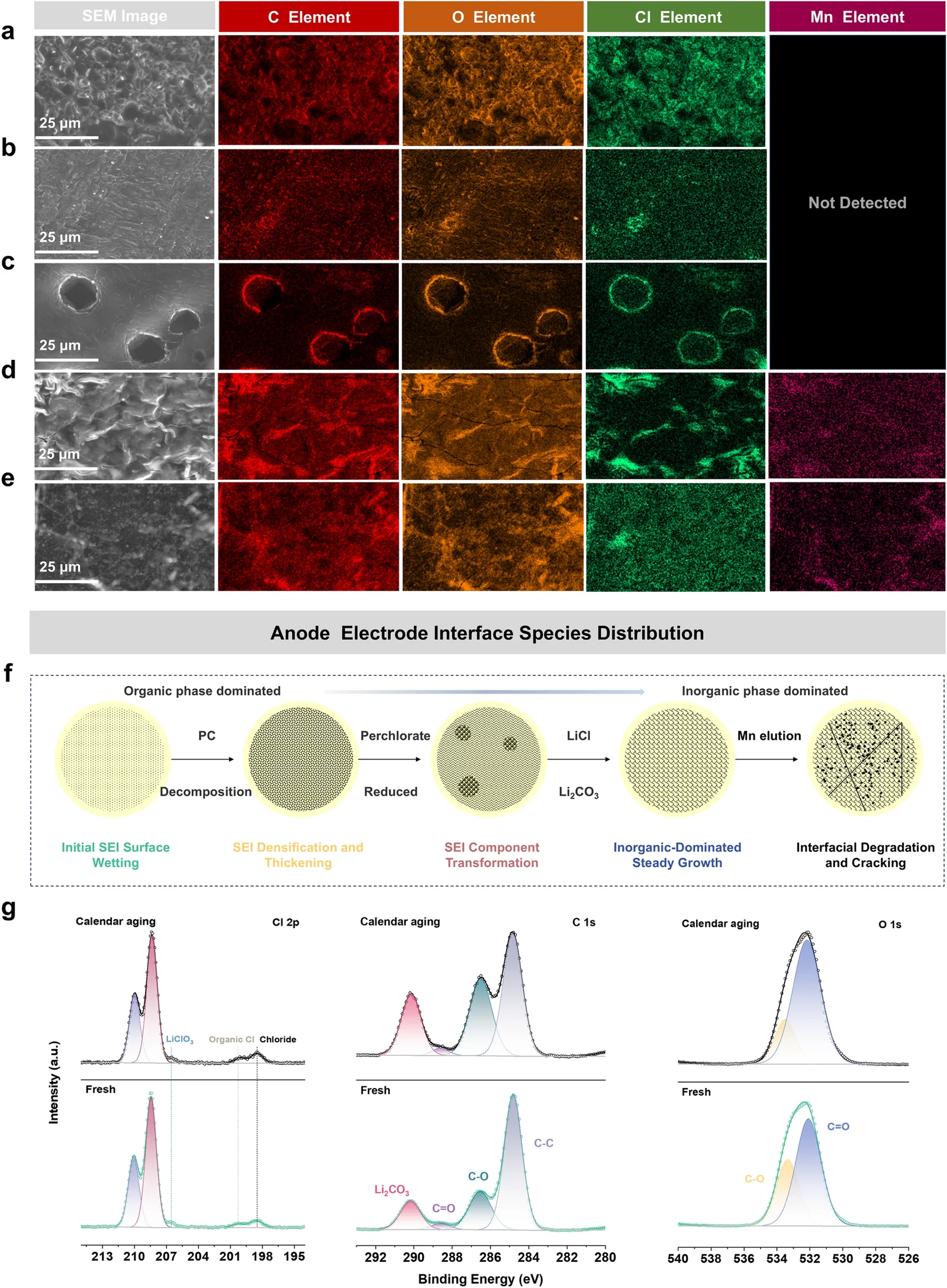
Structural and chemical characterizations of postmortem anode electrode with different calendar time. The SEM and EDS images of postmortem anode electrode at stage of** a** fresh, **b** 3 years, **c** 6 years, **d** 8 years, **e** 10 years. **f** Postmortem anode electrode interface species distribution diagram. **g** Cl 2*p*, C 1*s*, and O 1*s* XPS spectra of postmortem anode electrode

To elucidate the interfacial degradation mechanism of the anode in Li-MnO_2_ primary batteries during calendar aging, SEM, EDS, and XPS were performed on Li metal anodes after different storage durations, as presented in Fig. 4. Regarding the physical surface of the postmortem lithium metal, the original lithium anode showed a relatively flat surface, with only the inherent microtexture of the original lithium foil (Fig. S3a), while the surface of the 10-year anode electrode produced obvious yellow plaques (Fig. S3e). SEM images show that the initial solid electrolyte interface (SEI) is amorphous and gradually densified (Fig. S3f, g). With prolonged storage, the electrolyte continues to undergo continuous reduction side reactions on the surface of lithium, and the cross-sectional morphology is crater-like, indicating that the SEI component gradually changes from organic to inorganic. In the later stage of storage, the SEI was thickened and brittle and the surface was attached with small particles. This evolution follows the traditional SEI growth kinetics. The growth rate is fast in the early stage and gradually slows as the mass transport resistance through the film increases, yet never fully ceases due to persistent grain boundaries and microdefects in the polycrystalline SEI.

EDS elemental mapping provides direct visualization of the compositional evolution at the anode interface. Mn is undetectable in pristine and medium-term aged samples, while distinct Mn distribution appears on the long-term aged anode surface (Fig. 4a–e). This confirms that trace amounts of Mn ions eluted from the MnO_2_ cathode during calendar aging, migrate through the electrolyte, and are reductively deposited on the anode. The dissolved transition metal introduces defect sites in the SEI and catalyzes further electrolyte decomposition, accelerating SEI growth and interfacial degradation. Based on the morphological and elemental evidence, the SEI evolution process for calendar-aged Li anodes is proposed (Fig. 4f). In the initial stage, the lithium metal contacts with the electrolyte to spontaneously form a thin SEI film dominated by organic matter. In the early aging stage, rapid reduction of PC solvent and LiClO_4_ salt leads to fast densification and thickening of the SEI. Component reconstruction occurs in the middle stage. Unstable organic components such as alkyl carbonates are gradually converted into thermodynamically more stable inorganic Li2CO_3_ and LiCl, driving the transition of the SEI from organic-dominated to inorganic-dominated. In the long-term aging stage, the SEI enters a steady slow-growth period dominated by inorganic phases. After ultra-long storage, the excessively thickened SEI becomes brittle; coupled with the catalytic effect of deposited Mn, microcracks and local degradation emerge in the interface, allowing continuous electrolyte penetration and sustained side reactions along the defect channels.

XPS results validate the above evolution law at the chemical state level (Fig. 4g). The Cl 2*p* spectra reveal that Cl species in the pristine sample are dominated by undecomposed ClO_4_^−^ with only a tiny fraction of reduced Cl^−^. After calendar aging, the relative peak area of chloride increases markedly, evidencing continuous reductive decomposition of the lithium salt during storage, with LiCl as the stable reduction product. The C 1*s* spectra are deconvoluted into three characteristic peaks. The peak at 284.8 eV is assigned to C–C bonds while the peak at 286.5 eV corresponds to C–O single bonds from alkoxide organic reduction products, and the peak at 290.2 eV is attributed to Li_2_CO_3_ and alkyl lithium carbonates. After calendar aging, the relative intensity of the peak assigned to Li_2_CO_3_ increased significantly, confirming the continuous enrichment of inorganic carbonate components in SEI.

### Postmortem Electrode Interface Evolution Analysis

**Fig. 5:**
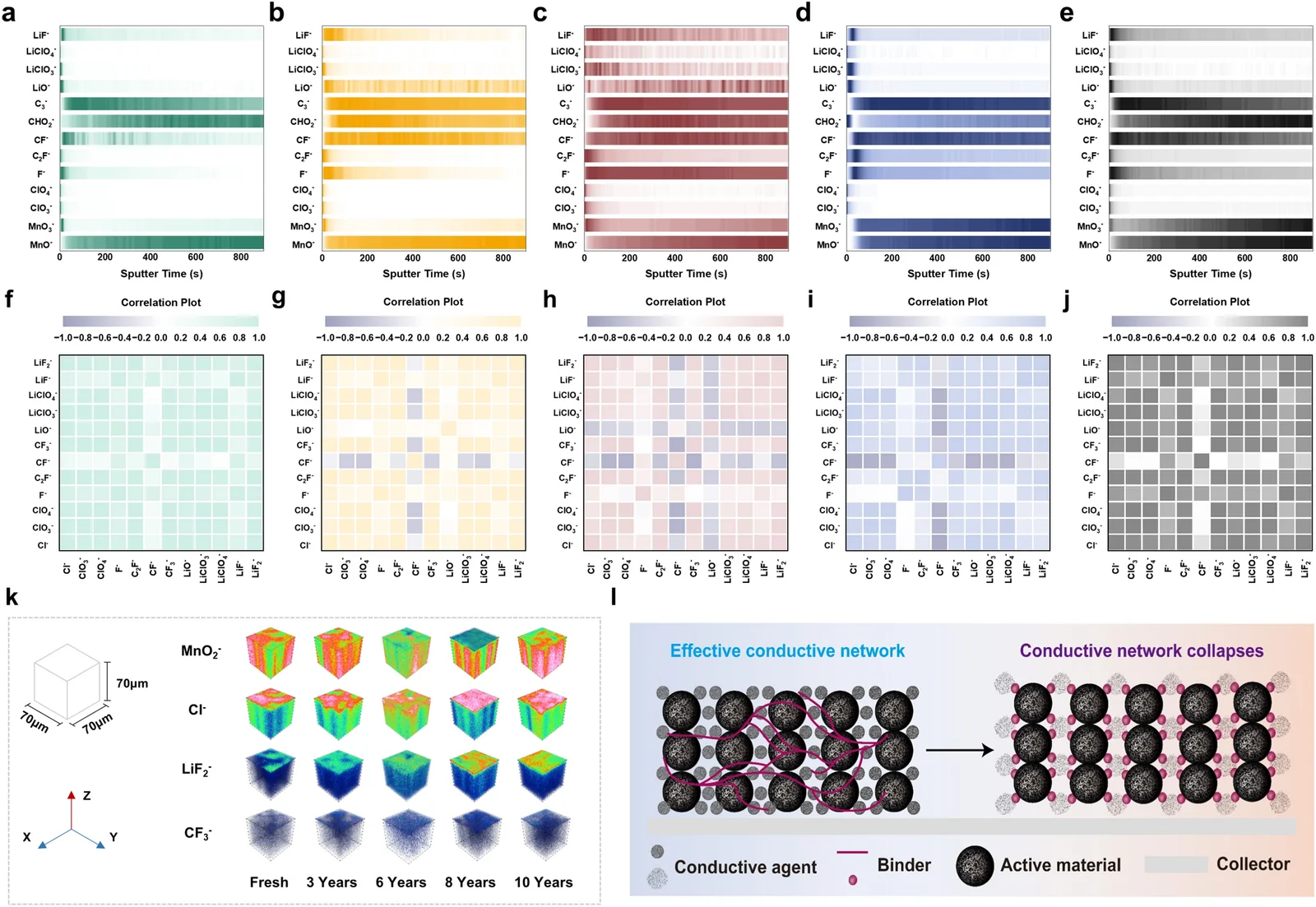
Characterization of ToF-SIMS to identify ion fragments under different calendar time. Depth profiling of postmortem cathode electrode of **a** fresh, **b** 3 years, **c** 6 years, **d** 8 years, **e** 10 years. Correlation matrix of selected ion fragments on postmortem cathode electrode of **f** fresh, **g** 3 years, **h** 6 years,** i** 8 years, **j** 10 years. **k** 3D composition of postmortem cathode electrode of different calendar time.** l** Schematic diagram of interface evolution under calendar aging

To further investigate the effect of the electrolyte on the interfacial chemistry and structure of postmortem cathode, specific secondary ion fragments were analyzed, which were identified as representatives of degradation species from the electrode and electrolyte. Ion mixing beam effects were excluded during the analysis, and fragments as well as recombination between species with low ion flux were neglected. Figure 5a–e shows the negative ion fragments considered as intermediate products. LiClO_4_^−^, LiClO_3_^−^, LiO^−^, CHO_2_^−^, ClO_4_^−^, and ClO_3_^−^ correspond to the primary and secondary derived fragments of the electrolyte and solvent. The intensities of fragments were most prominent with increasing sputtering time during the middle storage stage, subsequently, the intensities of these intermediate products gradually decreased as they transformed into more stable deposits. F-containing fragments indicate the inevitable degradation of PTFE during storage, and the degraded PTFE then combines with Li^+^ to form LiF, which aggravates interfacial impedance. The derived fragments of MnO_2_, namely MnO and MnO_3_^−^ species, are attributed to redox reactions in the local chemical environment. Combined with the previous XPS results, the composition of the CEI exhibits dynamic changes during storage. It is noteworthy that interfacial components such as organolithium salts, fluorine-containing compounds, and chlorides can provide a stable self-discharge barrier. However, the subsequent side reactions cannot be suppressed in an environment with high Li^+^ concentration and under the intrinsic catalytic properties of MnO_2_, which severely impairs the C-rate performance of the cells.

To establish a detailed correlation between the interphase chemical structure and cell performance, a further analysis was conducted on the correlations among various secondary ion fragments representative of CEI. Higher negative ion correlation in postmortem electrode indicates a chemically homogeneous CEI. As shown in Fig. 5f–j, the color-coded correlation matrices of the samples were generated from depth-integrated ToF-SIMS maps, with target fragments normalized by the range along the depth profile. The fragments related to PTFE, electrolyte, solvent, and lithium salts exhibited a high positive correlation among groups of fresh cells. With the extension of storage time, the PTFE main chain generated CF^−^ fragments with the highest abundance under high-energy ion sputtering. The CF^−^ fragments typically showed a negative correlation with other degradation products, which was most prominent at the 6th year of storage. Subsequently, the homogeneity among various fragments slightly improved, which is associated with the re-consumption of intermediate products to form stable lithium salts.

To visualize the spatial distribution of key chemical fragments, 3D render of the samples was created. The decreased signal intensity of the MnO_2_ surface layer is associated with the generation of MnO^−^ and MnO_3_^−^ species. Fortunately, the impact on the bulk phase is minimal, exerting only a slight influence on the capacity at low current density. Cl^−^ is indicative of electrolyte decomposition, while CF_3_^−^ fragments imply that the main structure of PTFE remains intact. However, the generated fragments irreversibly form LiF^−^ and LiF_2_^−^ species. The dynamic transformation of CEI components severely exacerbates interfacial impedance.

**Fig. 6:**
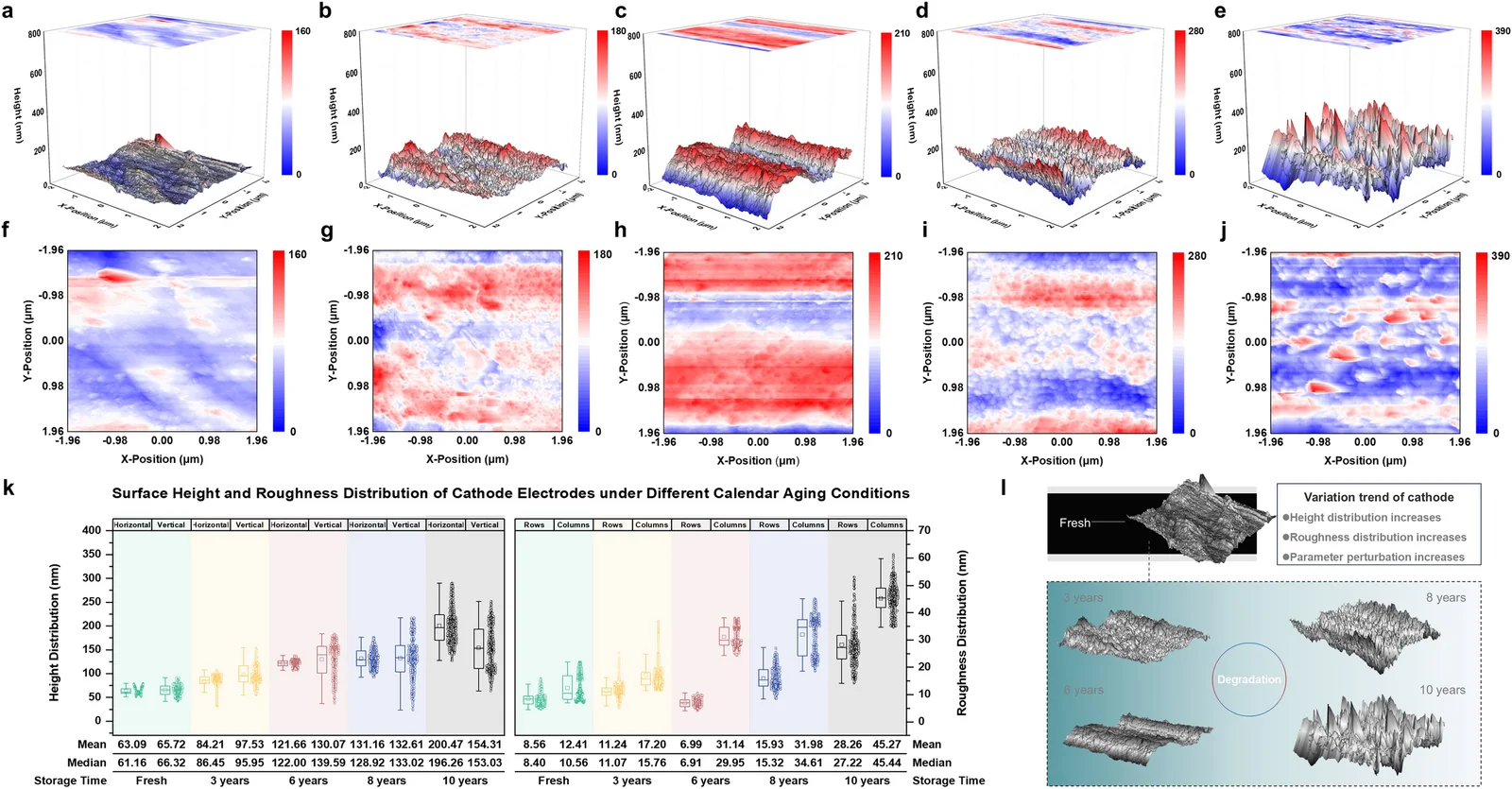
Characterization of AFM to identify height and roughness of postmortem electrode under different calendar time. AFM images of **a, f** fresh, **b, g** 3 years, **c, h** 6 years, **d, i** 8 years, **e, j** 10 years. **k** Surface height and roughness distribution of postmortem electrode of different calendar time. **l** Schematic diagram of interface height and roughness change under calendar aging

Atomic force microscopy (AFM) was employed to analyze the dynamic evolution of surface height and roughness of postmortem electrode during storage, facilitating an in-depth understanding of the reaction kinetics at the electrode interface. Figure 6a–j presents the AFM images of postmortem cathode electrode under different calendar aging durations, acquired at a scale of 2 μm × 2 μm. The height variations in the horizontal and vertical directions, plotted with the (0, 0) coordinate as the center, show that the surface height of fresh cell electrodes is relatively uniform. Subsequently, the surface height increases continuously with random distribution, which is consistent with the SEM observations. Statistical analysis was performed on the AFM height and roughness datasets. The vertical average heights of the electrodes from fresh, 3-, 6-, 8-, and 10-year stored cells are 65.72, 97.53, 130.07, 132.61, and 154.31 nm, respectively. Correspondingly, the columns mean roughness (R_a_) values for the five states are calculated to be 12.41, 17.20, 31.14, 31.98, and 45.27 nm, respectively (Fig. 6k). The postmortem cathode of the fresh cell exhibits the lowest surface height distribution and roughness, which is attributed to the high quality surface solvent wetting state and the effective inhibition of the initial pre-discharge formation of CEI. Therefore, the surface smoothness in different directions is lower than that of electrodes after calendar aging. Overall, the initial CEI generated by the pre-discharge step in commercial lithium primary battery manufacturing effectively suppresses early-stage Mn dissolution and stabilizes the pristine electrode–electrolyte interface, yet it is not a fully inert passivation interface. It contains metastable organic constituents that undergo progressive compositional reconstruction over prolonged storage, evolving into high-resistance inorganic-rich species that drive persistent interfacial degradation.

**Fig. 7:**
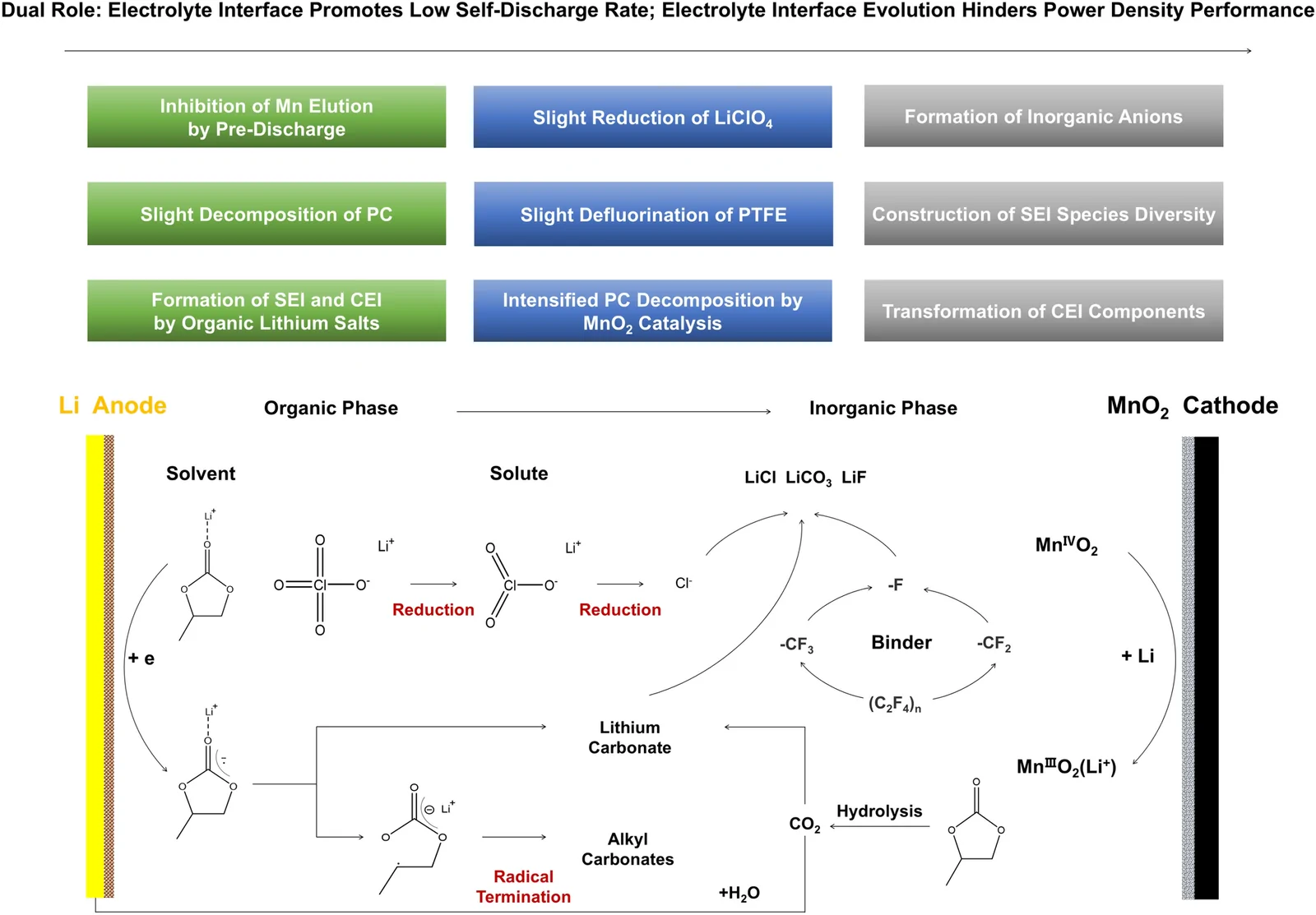
Internal evolution mechanism of Li-MnO_2_ battery under calendar aging

The multi-interfacial synergistic degradation mechanism for calendar-aged Li-MnO_2_ primary batteries is established (Fig. 7). On the lithium metal anode, PC is not only reduced to form alkyl carbonate intermediates, but also converted to thermodynamically stable Li_2_CO_3_ through the hydrolysis pathway. Simultaneously, the LiClO_4_ solute experiences stepwise deoxygenation reduction to form stable LiCl [48–50]. The as-formed initial SEI is dominated by organic lithium salts; with extended storage, metastable organic components progressively reconstruct into inorganic phases. On the MnO_2_ cathode, the intrinsic catalytic activity of MnO_2_ accelerates oxidative decomposition of the electrolyte, inducing dynamic compositional transformation of the CEI [51]. Meanwhile, gradual defluorination of the PTFE binder releases fluorine-containing fragments that react with mobile Li^+^ to form LiF, further raising interfacial impedance [52]. Accumulated insulating by-products on the cathode gradually block electron conduction pathways between active materials and conductive carbon, which is identified as the dominant contributor to the severe deterioration of high C-rate discharge capability. Additionally, trace dissolved Mn ions migrate to the anode and are reductively deposited, catalyzing further electrolyte reduction and forming a coupled cathodic–anodic degradation loop that accelerates interfacial deterioration in the long storage stage. The interfacial films exhibit a distinctive dual role: The passivation effect effectively suppresses bulk self-discharge, whereas their irreversible growth continuously increases charge-transfer impedance and aggravates high-rate polarization. The core contradiction governing calendar aging lies in the inherent dual nature of electrode–electrolyte interphases. Looking ahead, investigating storage characteristics across representative SOC levels would better mimic practical operating environments and yield more actionable insights into degradation pathways, which represents a valuable direction for future investigations.

## Conclusion

In summary, comprehensive performance analysis of lithium primary cells subjected to a 10-year sequential calendar aging process was systematically conducted, revealing the degradation mechanism applicable to various similar or homologous cells. Electrochemical performance, microstructure, and interface analysis were mainly carried out to describe the dynamic evolution process. Regrettably, although the self-discharge rate of the cells remained at a relatively low level under low current density, the capacity retention rate under high C-rate underwent severe nonlinear decay to 66.3%, accompanied by a significant reduction in consistency, which deviates from the typical Arrhenius relationship. Comprehensive analysis indicates that the electrolyte, binder, and active material undergo irreversible degradation, triggering intense side reactions during long-term calendar aging. The ionic fragments reflect a gradual transition from compositional diversity to stabilization during storage. Organolithium salts, fluorine-containing compounds, and chlorides as effective barriers contribute to the low self-discharge rate characteristic of lithium primary cells. Paradoxically, further deposition of these species at the interfaces between active materials and conductive agents significantly increases the electrode interfacial impedance, thereby deteriorating the C-rate performance. Based on this work, the disclosed dual-role mechanism of electrode–electrolyte interface is generalizable to other lithium primary and secondary batteries under long-term calendar aging, providing a reference for interfacial optimization and lifetime evaluation.

## Supplementary Information

Below is the link to the electronic supplementary material.

Supplementary file1 (DOCX 5819 KB)
