# Supplementary material for: Calendar Aging Triggers Critical Performance Degradation of Lithium Primary Batteries
Source: Nanomicro Lett. 2026 Nov 5;19:57. doi: 10.1007/s40820-026-02354-x (PMC13737069; doi:10.1007/s40820-026-02354-x)
Supplement: Supplementary file 1 [file 40820_2026_2354_MOESM1_ESM.docx]

Supporting Information for

**Calendar Aging Triggers Critical Performance Degradation of Lithium Primary Batteries**

Mengjie Li^1,2^, Shuzhi Zhao^3^, Fei Wei^1^, Wen Jia^1^, Zi-Feng Ma ^1^*

^1^ Shanghai Electrochemical Energy Devices Research Center, School of Chemistry and Chemical Engineering, Shanghai Jiao Tong University, Shanghai 200240, P. R. China

^2^ Shaanxi Applied Physics Chemistry Research Institute, Xi'an710061, P. R. China

^3^ College of Smart Energy, Shanghai Jiao Tong University, Shanghai 200240, P. R. China

^*^Corresponding author. E-mail: [zfma@sjtu.edu.cn](mailto:zfma@sjtu.edu.cn) (Zi-Feng Ma)

**S1 Basic information of battery**

The basic information and storage conditions of the battery are as follows. Nominal capacity: 3.4 Ah. Anode: Lithium metal foil, 0.1 mm in thickness. Cathode: MnO_2_ composite electrode, 0.3 mm in thickness, with an active material (MnO_2_) areal loading of 80 mg/cm². Cathode current collector: Porous aluminum mesh (enables higher active material loading than planar aluminum foil). The pre-rolling thickness is 0.4 mm, with an aperture size of 2 × 2.2 mm. Storage Conditions: The 10 years calendar aging was conducted via natural static storage in a standard professional battery warehouse. The ambient temperature was maintained at10-25°C with a relative humidity of 40%-75%. Cells with different aging durations were stored under identical conditions to ensure the comparability of characterization results.

**S2 Electrochemical characterization**

Cells from different batches were stored in a standard storage warehouse. The samples were labeled as initial state (fresh), 3rd year, 6th year, 8th year and 10th year according to the batch time. The electrochemical performances of the cells were characterized using a Chroma battery testing system, which included capacity measurement, C-rate performance evaluation, and galvanostatic intermittent titration technique (GITT) testing, with a cut-off voltage of 1.5 V. Electrochemical impedance spectroscopy (EIS) measurements were conducted on a CHI 660 electrochemical workstation over a frequency range of 100 kHz to 0.01Hz. All calendar aged cells were disassembled under an ambient dew point below -40 °C, and the electrodes were collected and stored for subsequent material characterization.

**S3 Postmortem electrode characterizations**

X-ray diffraction (XRD) patterns were recorded using a D8 Advanced X-ray diffractometer. Fourier transform infrared (FTIR) spectra in the range of 4000–500 cm⁻¹ were acquired with an M2000 FTIR spectrometer. X-ray photoelectron spectroscopy (XPS) was performed on the SHIMADZU Kratos AXIS Ultra DLD. The surface morphology of the cathode electrodes was examined using a TESCAN RISE-MAGNA field-emission scanning electron microscope (FE-SEM). High-resolution transmission electron microscope (HRTEM) images were captured by the FEI Talos F200X G2 TEM. AFM was performed (Keysight Technologies, 5500) to characterize the roughness and height of the cathode electrodes. Time-of-Flight Secondary Ion Mass Spectrometry (ToF-SIMS) measurements were performed on a TOF-SIMS 5-100 instrument (ION-TOF GmbH, Germany) in negative ion mode. Depth profiling was conducted using an interlaced mode with a 1 kV Cs⁺ sputtering beam (~15 nA, 45° incidence) rastered over a 280 × 280 μm area, and a 60 keV Bi_3_^2^⁺ analysis beam (0.45 pA, 45° incidence) scanned over a 70 × 70 μm area (128 × 128 pixels). A low-energy electron flood gun was employed throughout the measurements for charge compensation. The mass range was set to 0-910 amu to cover all relevant electrolyte decomposition products and binder fragments.

GITT applies a constant current I to a system at a certain time interval t, and measures the change in potential over time during the current pulse period. During the current pulse, constant lithium ions pass through the electrode surface and diffuse according to Fick's second law. The diffusion coefficient of lithium ions can be obtained by the following formula, ignoring the slight volume change of electrode particles when lithium ions are embedded.

 (S1)

where τ represents the relaxation time, m_B_ denotes the mass of the active material, V_m_ is the molar volume of the active material; M_B_ is the molar mass of the active material, S is the electrode area, ΔE_s_ is the voltage change caused by relaxation process, ΔE_t_ is the voltage change caused by the pulse process, and L is the thickness of the electrode.

**S4 Supplementary Figures**

**Fig. S1** XRD pattern of MnO_2_, Super P Li and PTFE.

**Fig. S2** FTIR spectra of MnO_2_, Super P Li and PTFE.

**Fig. S3** The photographs of postmortem anode electrode at stage of (a) fresh, (b) 3 years, (c) 6 years, (d) 8 years, (e) 10 years. SEM images of (f, k) fresh, (g, l) 3 years, (h, m) 6 years, (i, n) 8 years, (j, o) 10 years.

**Fig. S4** 3D composition of postmortem cathode electrode (Fresh and 10 years cathode).

**Fig. S5** AFM raw data of postmortem electrode with different calendar time.

**Fig. S6** Surface height distribution of postmortem electrode of different calendar time (a) fresh, (b) 3 years, (c) 6 years, (d) 8 years, (e) 10 years.

**Fig. S7** Surface roughness distribution of postmortem electrode of different calendar

time (a) fresh, (b) 3 years, (c) 6 years, (d) 8 years, (e) 10 years.
